# SARS-CoV-2 variant omicron XBB.1.5: challenges and prospects – correspondence

**DOI:** 10.1097/JS9.0000000000000276

**Published:** 2023-03-13

**Authors:** Nikhil K. Channabasappa, Ankush K. Niranjan, Talha B. Emran

**Affiliations:** a Department of Veterinary Physiology and Biochemistry; b Department of Veterinary Microbiology, College of Veterinary Science and Animal Husbandry, Rewa, Madhya Pradesh, India; c Department of Pharmacy, BGC Trust University Bangladesh, Chittagong; d Department of Pharmacy, Faculty of Allied Health Sciences, Daffodil International University, Dhaka, Bangladesh

Highlights

- The XBB.1.5 variant accounts for less than 10% of coronavirus disease 2019 cases.
- The XBB.1.5 is a recombinant of two descendants from the BA.2 lineage.
- The XBB.1.5 variant is thought to have a greater affinity to the ACE2 receptor.
- 1.5 is derived from the omicron variant BA.2.

*Dear Editor,*

For the past 2 years, the world has been witnessing the unprecedented ongoing pandemic caused by coronavirus disease 2019 (COVID-19). Even after the development of multiple vaccines, the virus is still causing havoc due to the emergence of new variants. This is an alarming situation to the scientific community that the virus is not over yet, looming around us causing disaster one after the other. The XBB.1.5 variant, which accounts for less than 10% of COVID-19 cases in the United States of America (USA), is now responsible for more than 25% of cases in a short span of 4 weeks. According to the experts, people already infected with an earlier omicron variant are likely susceptible to re-infection with XBB.1.5 due to enhanced immune evasion in comparison to other strains and waning immunity over time.

The XBB.1.5 is a recombinant of two descendants from the BA.2 lineage that caused disaster in early 2022, with a further spike in RBD mutation S486P (Fig. 1). This lineage was first detected in the USA from the samples collected on 22 October 2022, and since then the variant is raising the global concern. As of 9 January 2023, a total of 4770 sequences have been submitted belonging to XBB.1.5 with the mutational profile in spike regions viz. Q183E, F486P, and F490S. Additionally, the F486P mutation is mainly responsible for the phenotypic change of the variant XBB.1.5. This mutation was rare during the earlier pandemic, as it requires two nucleotide substitutions in the same codon to change from phenylalanine to proline^1^. Further, from 22 October 2022 to 11 January 2023, a total of 5288 sequences of the Omicron XBB.1.5 variant have been reported from 38 countries. Most of these sequences are from the USA (82.2%), United Kingdom (8.1%), and Denmark (2.2%), and the variant has also been detected in several other European countries like Austria, Belgium, Czechia, Portugal, Romania, Slovenia, Denmark, Ireland, Italy, France, Germany, the Netherlands, Spain, Iceland, and Sweden (https://www.ecdc.europa.eu/en/news-events/update-sars-cov-2-variants-ecdc-assessment-xbb15-sub-lineage). WHO’s Technical Advisory Group on Virus Evolution met on 5 January 2023 to assess the risk associated with Omicron XBB.1.5. On the basis of genetic characteristics and early growth rate estimates, the committee concluded that the variant might contribute to increases in case incidence. To date, the overall confidence in the assessment is low as growth advantage estimates are only from one country, the USA^2^.

**Figure 1:**
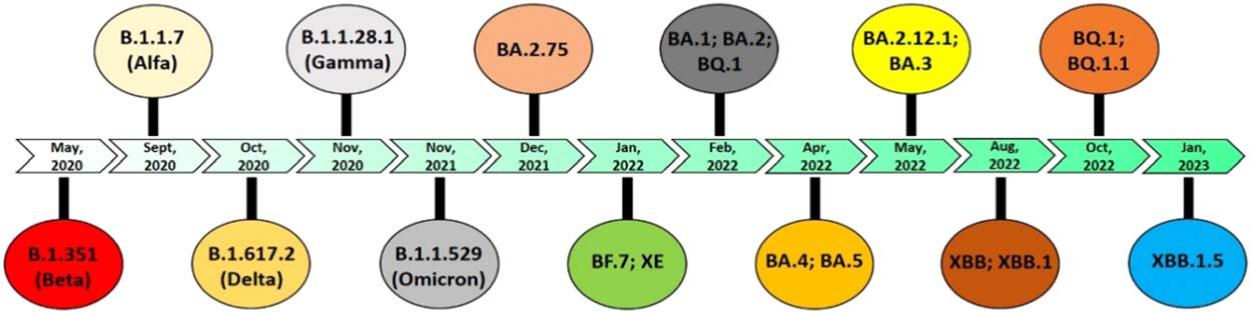
Time line describing the major variants, subvariants, and recombinant variants of severe acute respiratory syndrome coronavirus 2.

Surprisingly, Eric Feigl-Ding warned that there is a new recombinant strain XBB.1.5 in the scene, which is likely to become the ‘next big thing.’ Now the same is causing havoc in the USA due to an instant surge in the number of hospitalizations. He also said that multiple models show that the XBB.1.5 variant is much worse in transmission, *R*-value, and infection rate than previous variants. The XBB.1.5 variant is thought to have a greater affinity to the ACE2 receptor resulting in an increased uptake of the virus by the cells and enhanced transmissibility. This variant evades protective antibodies as effectively as the XBB.1 and is also better at binding to the cells^3^. The significant difference between XBB.1.5 and other variants is that it is one of the most immune-resistant variants to date and has the potential to prove very costly to the world population in the near future. ‘Ironically, probably the worst variant that the world is facing right now is actually XBB’ said Dr Michael Osterholm, an infectious disease expert at the University of Minnesota, in an interview with Reuters.

Although XBB.1.5 does not carry any mutation known to be associated with a potential change in severity such as S:P681R^4^. Lab studies suggest that the bivalent vaccine is still effective in protecting against severe disease, perhaps not as much against infection. XBB.1.5 is derived from the omicron variant BA.2, and while the current bivalent vaccine was developed for the BA.5 variant, it has been shown to generate antibodies that recognize BA.2. Fascinatingly, throughout 2022, researchers had a keen observation regarding the Omicron lineages picking up a series of antibody-evading mutations, especially in the viral spike protein that allowed new lineages to overcome immunity gained from vaccines and previous waves. Therefore, there is an urgent need to be better prepared for future variants because the virus is continuously evolving under selective pressure. Most of the vaccines are restricted to spike protein; there are certain chances that these spike protein-based vaccines may be ineffective against future variants to come. So, the development of bivalent, trivalent, or multivalent vaccine constructs or live vaccines could be the answer to future threats of SARS-CoV-2 (severe acute respiratory syndrome coronavirus 2).

## Ethical approval

Not applicable.

## Sources of funding

No funding was received.

## Author contribution

N.K.C.: data curation and writing – original draft preparation, reviewing, and editing; A.K.N.: conceptualization, data curation, and writing – original draft preparation, reviewing, and editing; T.B.E.: writing – reviewing and editing, visualization, and supervision.

## Conflicts of interest disclosure

The authors declare that they have no conflicts of interest.

## Research registration unique identifying number (UIN)

1. Name of the registry: not applicable.
2. Unique identifying number or registration ID: not applicable.
3. Hyperlink to your specific registration (must be publicly accessible and will be checked): not applicable.

## Guarantor

Talha Bin Emran, PhD, Associate Professor, Department of Pharmacy, BGC Trust University Bangladesh, Chittagong 4381, Bangladesh. Tel: +88 030 335 6193, fax: +88 031 255 0224. https://orcid.org/0000-0003-3188-2272.

## Data availability

The data in this correspondence article is not sensitive in nature and is accessible in the public domain. The data is, therefore, available and not of a confidential nature.

## Provenance and peer review

Not commissioned, internally peer-reviewed.
